# The Biomechanics of Healthy Older Adults Rising from the Floor Independently

**DOI:** 10.3390/ijerph20043507

**Published:** 2023-02-16

**Authors:** Elissa Burton, Keith D. Hill, Paul Davey, Yoke Leng Ng, Sîan A. Williams

**Affiliations:** 1 Curtin School of Allied Health, Curtin University, Perth, WA 6102, Australia; 2 enAble Institute, Curtin University, Perth, WA 6102, Australia; 3 Rehabilitation Ageing and Independent Living (RAIL) Research Centre, Monash University, Frankston, VIC 3800, Australia; 4 National Centre for Healthy Ageing, Monash University and Peninsula Health, Frankston, VIC 3199, Australia; 5 Curtin School of Nursing, Curtin University, Perth, WA 6102, Australia; 6 Health and Social Sciences Cluster, Singapore Institute of Technology, Singapore 138683, Singapore; 7 Liggins Institute, University of Auckland, Auckland 1010, New Zealand

**Keywords:** ageing, falls, three-dimensional movement analysis, up from floor

## Abstract

A third of older adults will fall each year and many will not be injured. Getting up from the floor in a timely manner is important, however it is unclear what technique older adults use to get themselves up off the ground unassisted, whether there are differences between men and women in getting up and what functional joint kinematics are used to rise from the floor. This study included a convenience sample of 20 older adults (65+ years) to answer these questions. Participants completed a series of movement tasks (i.e., rising from the floor using their own technique, a specified technique, walking 10 m and five repeated sit-to-stands), with temporospatial and joint kinematic data captured using an 18-camera 3D Vicon motion analysis system. Results found three techniques preferred by participants; the sit-up (n = 12), side-sit (n = 4) and the roll over (n = 4), with no differences found between sexes. The sit-up technique requires a higher degree of hip and knee flexion to complete compared to the side-sit and roll over. It may be beneficial for health professionals to work with older adults to identify their preferred technique for rising from the floor and encourage regular practice of this skill.

## 1. Introduction

Older populations around the world are increasing at rates not seen previously, and by 2050, the number of older adults is estimated to more than double worldwide from 727 million in 2020 to 1.5 billion [1]. The majority of older adults want to live in their own homes until they die, and to achieve this, they need to maintain their independence and ability to undertake activities of daily living, such as showering, dressing and feeding [2]. The risk of falls is also a considerable issue for many older adults. One in three people aged 65 and over living in the community will fall each year and this increases to 50% for those aged 80 years and over [3]. Many of those who fall will be injured [4], but there is also a proportion that fall who, although they are not injured, cannot get themselves up off the ground after the fall [5].

Helping a person up off the floor in a timely manner is essential for their longevity and long-term health. Those left lying on the floor for over an hour (i.e., considered a long lie), even without injury, are more likely to die within 6 months [6]. In a systematic review, Ebben and colleagues [7] found that between 11–56% of older adults who called an ambulance due to a fall did not need a transfer to hospital for medical assistance, nor did they require a referral for assistance after the episode. This scenario within falls-related calls for emergency services is referred to as a ‘lift-assist’ and is becoming an increasingly frequent occurrence. Data from a decade of emergency services calls from a community in Minnesota showed an increase in falls-related calls over the time period, but with proportionate decreases in those requiring transportation to hospital, with significant increases in ‘lift-assists’ [8]. Lift-assists were estimated to cost US$1.5 million over a 10-year period [8]. Issues of ‘lift-assist’ are not isolated to a single country, Australia has also identified it as an issue [9], unfortunately one which currently receives little attention.

Fear of falling can also occur for many older people and studies have reported between 3% and 92% [10,11] have been estimated to have a fear of falling and 50% may experience fear of falling without having experienced a fall [12]. A fear of falling can lead to a withdrawal from activities, which may see a decline in function and an increased risk of future falls [13]. Studies have found those with a higher fear of falling are more likely to find it difficult to sit and rise from the floor [14,15].

The recently published world guidelines for falls prevention and management for older adults noted the importance of being able to get up from the floor independently, in particular, to avoid harm from long lies [16]. Exercise and physical activity are strongly recommended to reduce the risk of falling, with high evidence for balance and functional exercise programs [16]. They also recommend older adults practice the skill of getting up off the floor and evidence has shown that it is possible to regain this skill [16,17].

There are predominantly two main methods described in the research about how to teach an older adult to get up off the ground; the ‘backward chaining’ and ‘conventional method’ [18,19]. The backward chaining method, which is recommended to be used in the world guidelines [16], starts from standing and works through the process of getting onto the ground and then uses the same processes to rise up off the ground [18,19]. The conventional method requires the person to lie supine, roll on their side, support one arm, sit on the opposite buttock to the supported arm, turn to have both arms straight and hands on ground and transfer weight onto both knees, move into a half-kneeling position and push to an upright position [20]. These techniques seem to be widely taught, yet there is little evidence in understanding what technique older adults who can get up independently instinctively use themselves [21]. Also unknown is what functional joint ranges of motion are required to achieve the task of rising from the floor independently. This research aimed to fill this gap by answering the following research questions:

Is there a common/preferential movement pattern adopted by older adults to rise from the floor independently?

Are there any sex differences in the techniques used for rising-from-the-floor?

What functional joint kinematics are used to achieve the rising-from-the-floor task?

Are there associations between rising from the floor performance and physical assessments of sit to stand and walking speed?

## 2. Materials and Methods

### 2.1. Sample and Recruitment

A convenience sample of 20 older adults (aged 65+ years) were recruited from the community (via local community radio) and word of mouth for this cross-sectional study. For inclusion, participants needed to be living in the community and were able to get up off the floor multiple times without assistance (self-reported). Exclusion criteria were any pre-existing health conditions or injury that would make it unsafe to take part or being unable to rise-from-the-floor independently (self-reported). Informed written consent to participate in a protocol approved by Curtin University Human Research Ethics Committee (HRE2017-0714) was obtained for each participant prior to data collection.

### 2.2. Participants

The average age of the 20 participants (n = 12 females) was 73.5 years (±4.6, range: 66–81 years), with an average weight of 76.5 kg (±14.3), height of 166.3 cm (±10.8) and body mass index of 27.6 kg/m^2^ (±4.3, range: 20.1–35.5) (Table 1). Two-thirds of the participants were married (n = 13), 25% separated or divorced (n = 5), one widowed and one participant had never married. Those married were living with their spouse (65%, n = 13) and those who were not lived alone (35%, n = 7). Nine participants completed high school (45%), four completed a trade (20%) and six completed a university degree (30%), with one of these completing a post-graduate degree. Two participants (10%) reported having trouble walking but they did not use an aid for assistance, the other 18 (90%) reported no trouble walking. Only 20% (n = 4) had fallen in the past year and of those three were injured, but only one received medical attention for the injuries. Two-thirds of the group (n = 13) reported no pain in the 24 hours before testing, seven participants’ pain ranged from mild (n = 5) through to moderate (n = 2).

### 2.3. Procedure

Participants attended one data collection session at the Curtin University Motion Analysis Laboratory and were asked to wear a singlet and shorts (i.e., close-fitting clothes) to allow for the placement of retro-reflective markers. Following the completion of a demographic and health questionnaire (capturing age, sex, height, falls history in past 12 months, numeric pain rating scale—current pain, best and worst pain in last 24 h), participants were fitted with a full lower body 12.7 mm retro-reflective marker set at specified anatomical locations [22] for the three-dimensional motion analysis (3DMA) testing. Participants were then asked to complete a series of movement tasks, with temporospatial and joint kinematic data captured using an 18-camera 3D Vicon motion analysis system (Vicon, Oxford Metrics; 250 Hz). All movement trials were completed in the same order for all participants, were repeated a minimum of three times (i.e., three successful repeats of task execution) and were completed barefoot. Participants were given time to rest between tasks if required.

### 2.4. Movement Task: Walking Gait

Participants were asked to walk in a straight line for approximately 10 m at a self-selected (i.e., comfortable) pace [23]. Average walking speed was calculated from the velocity taken of the retro-reflective marker located on the posterior superior iliac spine.

### 2.5. Movement Task: Floor to Stand (FTS)

Participants began in a supine position on the ground and were asked to ‘stand up’. No instructions, advice or assistive equipment (e.g., a bench or box) were given, such that this was their own ‘self-selected’ method. This was repeated to determine consistency in approach.

Participants were then instructed on the ‘conventional’ method of FTS while using a box for assistance [19], through verbal instructions and a demonstration, and were allowed a practice before undertaking testing. This included starting in supine on the ground, rolling onto their preferred side, pushing up with their hands and knees, placing the closest hand onto a box, placing the strongest leg forward (i.e., one knee still on the ground), pushing up and standing up.

### 2.6. Movement Task: Sit-to-Stand Five Times

A trial of five repeated sit-to-stand (STS) time trials were completed using a box set to allow a starting position of 90° flexion at the hip and knee joints with feet firmly flat on the ground. Participants were instructed to cross their arms over the chest, if necessary, they could lean forward and were instructed to stand up, after standing fully, they returned to a seated position. This was repeated five times [24]. The time it took for the participant to complete this was taken; they were not instructed to do this as quickly as possible.

### 2.7. Analysis

Demographic data and associations (using Pearson product-moment correlation) between FTS (both ‘self-selected’ and ‘conventional’ methods) and walking speed and sit to stand (STS) were conducted using SPSS v27. Positive strength of association was considered weak, 0.1 to 0.3, moderate, 0.3 to 0.6, and strong, 0.5 to 1.0, negative strength of association was considered weak, −0.1 to −0.3, moderate, −0.3 to −0.5, and strong, −0.5 to −1.0 [25].

Motion analysis data were labelled, filtered (Woltering filter, 10 Hz; determined using residual analysis) and modelled using Vicon Nexus software and a customized Labview program 2018 SP1 (National Instruments, Austin, TX, USA) was used to model and output the data. For each participant, the average of the repeated trials was used to develop kinematic graphs (time normalized to 101 data points for comparison across trials), and the peak joint angles for each trial were extracted and, again, averaged for each participant. To determine the methods of FTS for participants, the timing of segmental motion was extracted from the 3D motion data to indicate the order of movement. Further to this, video data were visually inspected to confirm and describe each participants’ floor to stand method. Sex differences were analyzed using 2-sample *t*-tests.

## 3. Results

### 3.1. Self-Selected FTS Methods

All 20 participants demonstrated a preference in their method of FTS, with six trialing/demonstrating two methods (but still showing a preference for one method over another). Methods have been categorized by their Initiation movement: (i) Sit-Up (n = 12), (ii) Side-Sit (n = 4) and (iii) Roll-Over (n = 4), with multiple variations proceeding for Weight Transfer and Transition to Stand (Table 2). Time taken to complete each movement task is outlined in Table 2.

Thirteen participants favored their right side (n = 7 left). There was little difference between males and females for what technique they used for FTS. Differences did occur for the four participants (3 females, 1 male) who used the roll over technique. Two females progressed to half-kneeling (no male used this method) and one male and one female progressed to all fours (see Table 2 for more details).

**Figure 1:**
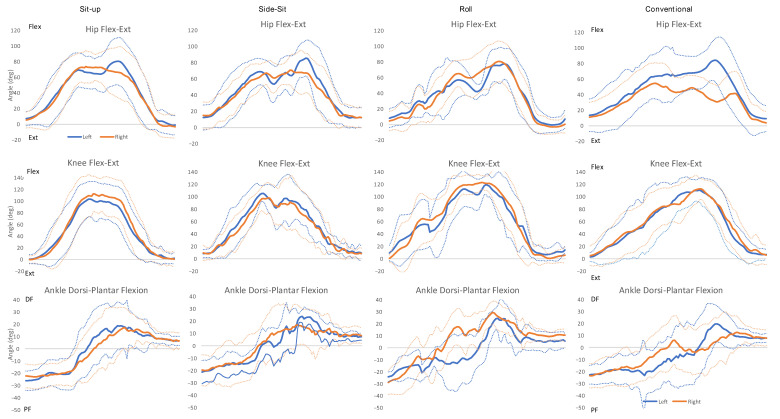
Kinematics graphs representing the average (solid lines) and standard deviation (dashed lines) of the sagittal plane for the hip, knee and ankle joints during the rise−from−floor tasks starting at supine, ending with standing. Data along the *X* axis plot the trajectory of the movement task, time-normalized to 101 data points, with 0 signifying the initiation of the task and 101 the completion.

Depending on the method, the mean maximum (peak) amount of hip flexion used to achieve the task ranged from 89.1° to 97.3°, and knee flexion ranged from 118.3° to 129.1°, with both ankle dorsi and plantar-flexion ranging between 27–33° (Table 3). Representative kinematic graphs of each method are presented in Figure 1.

### 3.2. Conventional FTS Method

**Figure 2:**
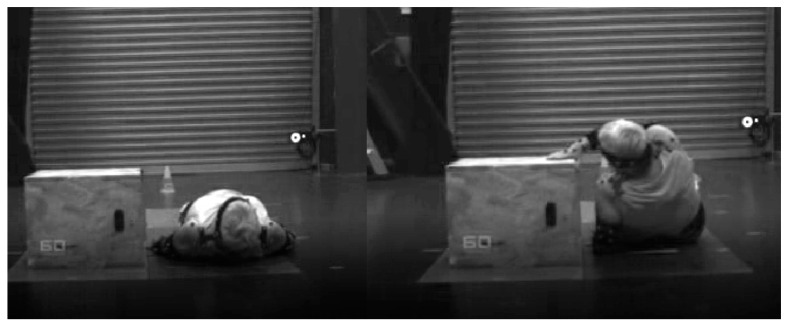
Participant using box to rise from the floor.

Peak hip, knee and ankle joint angles are also presented in Table 3, and Figure 1 presents a kinematic graph of the movement. A notable difference in the mean maximum (peak) amount of hip flexion was observed between limbs (Left: 100.4°, Right: 81.7°, *p* < 0.001), possibly relating to the position of the support box (Figure 2).

**Table 3:** Mean (±SD) and range data are presented for the peak sagittal kinematic angles at the hip, knee and ankle joints for each of the methods of the floor to stand task.

	Self-Selected Method			Conventional Method
Variable	Sit-Up	Side-Sit	Roll	
Peak Hip Flexion (°)	97.3 (11.2)	89.1 (19.6)	96.1 (16.3)	91.0 (15.5)
	71.5–120.7	56.3–117.1	72.9–129.9	64.2–120.1
Peak Knee Flexion (°)	129.1 (14.0)	118.3 (12.2)	126.0 (16.9)	124.1 (14.1)
	101.8–157.4	98.1–135.4	93.2–146.6	86.8–158.2
Peak Ankle Plantarflexion (°)	32.6 (10.3)	27.3 (10.3)	30.0 (6.0)	36.4 (7.6)
	9.5–48.5	12.8–44.1	20.6–37.6	21.6–50.8
Peak Ankle Dorsiflexion (°)	31.5 (7.6)	29.8 (7.8)	30.1 (6.2)	29.3 (7.1)
	9.2–44.1	18.8–42.2	21.7–43.2	18.6–45.4

### 3.3. Walking Gait Kinematics

Table 4 presents the peak sagittal kinematics of the hip, knee and ankle joints during one complete walking gait cycle and temporospatial parameters. Mean walking speed (m/s) for female participants was 1.22 (0.16) and 1.31 (0.13) for males.

### 3.4. Sit to Stand

Mean (SD) times are presented in Table 4. Mean sit-to-stand time (seconds) for female participants was 16.55 (3.55) and 14.47 (2.65) for males.

### 3.5. Associations between FTS and Walking Speed and Sit-to-Stand

A bivariate correlation was undertaken between FTS (for both the ‘self-selected’ and the ‘conventional’ methods) average times and walking speed (m/s) and the average duration for the STS. Results of the correlation indicate that there was a moderate negative correlation between ‘self-selected’ time to get up scores and walking speed (m/s) (r = −0.641, *p* = 0.003) and a moderate positive correlation with STS times (r = 0.563, *p* = 0.009). Results from the ‘conventional’ method time to get up scores showed a moderate positive correlation STS time (r = 0.546, *p* = 0.012), but there was no significant association with walking speed (m/s). There was also no association between walking speed and STS.

## 4. Discussion

This study found three different movement techniques that are preferred by older people when getting off the ground from a supine position, with no assistance or furniture. The most commonly used technique by 60% of participants was to begin by sitting straight up (like a sit-up), from this position a number of variations to get up off the ground were then used. The sit-up first technique was also the fastest to get to standing. Twenty percent of participants either used the side-sit or the roll-over technique, with the roll-over technique being the slowest of the three techniques for rising from the floor to standing. These findings share similarities with Bohannon and Lusardi [26]. However, they found that 50% (n = 26) of their participants used the side-sit to half-kneel pivot technique, 35% (n = 18) used the quadruped push-up and 15% (n = 8) used the sit-up and roll-over technique. The mean age of their participants was 64.6 (±9.5) years, they took 4.1 (±1.1) s to rise from the floor and their five-time-sit-to-stand was completed on average in 8.0 (±2.0) s [26]. These differences in technique may be due to their participants being on average 8.9 years younger, 2.3 s faster to get up off the ground using their own technique and 7.5 s faster to complete the five repeated sit-to-stand task. In another study, 53 older adults (mean age: 78.5 (±8.5) years) asked to rise from the floor used two particular techniques; 90.6% (n = 48) completed a partial roll and push maneuver and the other 9.4% (n = 5) used a symmetrical rise and asymmetric squat sequence [27]. Participants in this study took an average of 8.0 (±5.4) s to get up off the ground [27], which was slightly longer (1.6 s) than the current study. A final study by Ulbrich and colleagues’ [28] reported three different techniques for getting up from supine, the sit(-up), the crouch and the side lying; the average age of participants was 73 years and they completed the task in an average of 5.5 s, similar to this current study. It appears that the older the person, the more likely they are to use the roll over technique and be slower in getting up off the ground. This is likely due to a decrease in strength and balance; however, this was not directly measured in the current study. Health professionals teaching the skill of rising from the floor should also be aware that several different techniques are used by older adults to get up from the floor unassisted and no one technique is necessarily right for everyone. However, this is a skill that should potentially be practiced more frequently as people age to maintain technique, strength and independence should a fall occur.

Little difference was found between the sexes for techniques to get up, in particular the sit-up and side-sit techniques had the same number of males and females complete them. Two females using the roll-over technique progressed to half-kneeling which was not undertaken by any males. It is not clear why this difference occurred. Future research is required to better understand differences between the sexes and whether techniques being taught should be the same or not based on sex.

There is little research to date exploring the functional joint kinematics required to rise from the floor. Most studies investigating joint kinematics in older adults utilize walking, jumping, squatting and stair walking as activities [29,30,31,32,33,34]. However, two studies have looked at activities of daily living (ADLs) of older adults [35,36]. Hyodo and colleagues [35] explored 22 ADLs related to dressing, using the toilet, bathing, picking up objects and crouching. Getting out of the bath appeared to be the most similar activity to be able to compare to this current study. To get out of the bath the following was found: hip flexion 99° (±10°), knee flexion 143° (±8°), ankle plantar flexion 32° (±10°) and ankle dorsiflexion 28° (±7°), compared to our study for the self-selection technique where the following was found: hip flexion: sit-up: 97.3° (±11.2°), side-sit 89.1° (±19.6°), roll-over 96.1° (±16.3°); knee flexion: sit-up: 129.1° (±14.0°), side-sit 118.3° (±12.2°), roll-over 126.0° (±16.9°); ankle plantar flexion: sit-up: 32.6° (±10.3°), side-sit 27.3° (±10.3°), roll-over 30.0° (±6.0°); and ankle dorsiflexion: sit-up: 31.5° (±7.6°), side-sit 29.8° (±7.8°), roll-over 30.1° (±16.2°). Interestingly, greater knee flexion was required for getting out of a bath than standing up from the floor, but hip flexion, ankle plantar and dorsiflexion were within a few degrees difference between the two activities. Sah [36] used wearable sensors to measure different daily activities and reported a maximum average of 112.6° hip flexion for getting off the toilet, considerably more than this current study and the study by Hyodo and colleagues [35], who used an electromagnetic three-dimensional tracking system. For health professionals training in the skill of getting off the floor, range of movement limitations, pain and biomechanical factors that may be limiting a person’s ability to get up need to be taken into account when discussing potential techniques with their patient.

There is limited research that has investigated the associations between rising from the floor and a commonly used physical performance measure, the five-times-sit-to-stand. Two studies [26,37] were found, however one did not use average scores for each measure, but grouped participants for the sit-to-stand test into unable to achieve, poor performance (>13.6 s) and good performance (≤13.6 s) and for rising from the floor into independent, assisted and dependent groups [37], making comparisons more difficult. Bohannon and Lusardi [26] did collect these measures, with a mean time of 8.0 (±2.0) s for the five-time-sit-to-stand test and 4.1 (±1.1) s for the rising from the floor test. The Pearson correlation score for five-time-sit-to-stand and rising from the floor for the Bohannon and Lusardi [26] study was (r = 0.64, *p* = 0.001) similar to the current study’s self-selected technique and five-time-sit-to-stand (r = 0.563, *p* = 0.009), despite the difference in age described earlier between the studies. As people age, their ability to get up off the floor and to stand up from a chair five times (a measure for mobility) slows, and it is therefore important to promote strength and balance training regardless of age to ameliorate this. Studies have shown that strength can be improved at any age and it is important to promote this with older populations.

There were limitations for this study. Firstly, the conventional method of rising from the floor was not familiar to the participants and therefore it is possible that the kinematics could change over time with practice, we also did not measure muscle strength, muscle activation or kinetic analysis to explore the underlying mechanical forces or stressors on the joints and future studies should consider this. The study was also not sufficiently powered to detect significant associations between the floor rise and the physical assessments because this was not the primary aim of the study, future studies should consider this.

## 5. Clinical Implications

(1) Falls are common among older people, and a moderate proportion have long lies after their fall, which is associated with increased mortality and morbidity; and (2) health professionals should not only consider a single approach to training rising from the floor (either as a maintenance activity, or to work to regain ability to perform this task after an acute health event such as a stroke). Instead, the decision regarding which training approach to utilize should be individualized and be dependent upon the preference of the older person, as well as considering any biomechanical factors that may limit a person’s ability to perform one or more of the floor to rise approaches.

## 6. Conclusions

This study found older adults asked to rise from the floor to standing unassisted used three general techniques when not provided any instructions regarding technique. The most common technique started with completing a sit-up, and there was no difference in preferred approach between males and females. The sit-up technique requires a higher degree of hip and knee flexion to achieve compared to the side-sit and roll-over techniques. Moderate strength of association was found between both techniques (standardized or self-selected) for rising from the floor and sit-to-stand and the self-selected floor to rise method and walking speed. Health professionals are recommended to work with their older patients to identify their preferred method for rising from the floor unassisted and encourage regular practice of this skill.

## Figures and Tables

**Table 1:** Participant Characteristics.

Variable	Total Sample
Age M (SD)	73.5 (4.6)
Gender (Males) % (n)	40.0 (8)
Body Mass Index (BMI) M (SD)	27.6 (4.3)
Marital Status % (n)	
Never Married	5.0 (1)
Married/de Facto	65.0 (13)
Widowed	5.0 (1)
Separated/Divorced	25.0 (5)
Living Status % (n)	
With Spouse	65.0 (13)
Alone	35.0 (7)
Education Level % (n)	
High School	45.0 (9)
Trade/TAFE	20.0 (4)
Tertiary Education	30.0 (6)
Post-graduate Education	5.0 (1)
Chronic Health Conditions (Yes) % (n)	
None	15.0 (3)
High Blood Pressure	40.0 (8)
High Cholesterol	30.0 (6)
Cardiovascular	15.0 (3)
Prostate	10.0 (2)
Osteoarthritis	20.0 (4)
Fracture (Last 12 Months)	5.0 (1)
Inactive Thyroid	15.0 (3)
Type 2 Diabetes	5.0 (1)
Vision Issues	10.0 (2)
Gout	5.0 (1)
Irritable Bowel Syndrome	5.0 (1)
Number of Medications Prescribed M (SD)	2.7 (2.6)
Falls in Last 12 Months M (SD)	1.7 (0.6)
Current Pain Levels % (n)	
No Pain	65.0 (13)
Mild Pain	25.0 (5)
Moderate Pain	10.0 (2)

n: number of participants. M: mean. SD: standard deviation. TAFE: Technical and Further Education.

**Table 2:** Movement strategies demonstrated by participants from the Floor-to-Stand tasks, categorized by three stages.

Initiation	Weight Transfer	Transition to Stand	Participant Characteristics
Sit-up (n = 12) ^#^	Roll-over to push up position ^ (n = 4)	Rise from all fours (n = 3)	6F, 6M Age: 73.2 years (±4.8) Mean time to complete task: 5.98 s (±1.54) Walk-speed (m/s): 1.29 m/s (±0.16) STS time: 16.9 s (±3.5)
		One hand push to rise (n = 1)	
	Roll-over to one-knee on the ground (n = 1)	Rise from one-knee forward (n = 1)	
	Roll-over to both knees down (n = 5)	Rise from all fours (n = 2)	
		One-knee forward to rise (n = 2)	
		One-knee forward, one hand push to rise (n = 1)	
	Half-kneel pivot (n = 2)	Rise from all fours (n = 1)	
		One-knee forward to rise (n = 1)	
Side-Sit (n = 4)	All fours (n = 3)	Rise from all fours (n = 2)	2F, 2M Age: 73.5 years (±5.7) Mean time to complete task: 6.91 s (±0.69) Walk-speed: 1.02 m/s (±0.18) STS time: 14.5 s (±3.1)
		One-knee forward, one hand push to rise (n = 1)	
	Half-kneel pivot (n = 1)	Rise from all fours (n = 1)	
Roll-over (n = 4)	All fours (n = 2)	One-knee forward, one hand push to rise (n = 1)	3F, 1M Age: 73.7 years (±3.8) Mean time to complete task: 7.52 s (±4.35) Walk-speed: 1.29 m/s (±0.16) STS time: 14.4 s (±4.3)
		One-knee forward to rise (n = 1)	
	Half-kneel pivot (n = 2)	One-knee forward, two hands push to rise (n = 1)	
		Rise from all fours (n = 1)	

^ Knees off the ground; ^#^ One participant first raised knees up to chest for momentum to move into sit-up; m/s: meters per second; n: number of participants; F: female; M: male: STS: Sit-to-stand.

**Table 4:** Participants’ time performance on movement tasks, temporospatial parameters and average of peak sagittal kinematics through walking gait.

Timed Measure	Mean (±SD)	Range
Self-selected FTS (s)	6.41 (2.04)	4.32–12.50
Conventional FTS (s)	9.36 (4.02)	4.53–12.64
5-cycle STS (s)	15.5 (3.59)	10.9–22.1
Walking speed (m/s)	1.22 (0.18)	0.81–1.52
3DMA walking gait parameters		
Stride length (cm)	131.1 (19.7)	100.2–173.1
Stride width (cm)	10.1 (3.3)	3.8–18.1
Swing: Peak Hip Flexion (°)	36.0 (14)	13.5–69.5
Stance: Peak Hip Flexion (°)	34.8 (14.8)	7.4–69.7
Stance: Peak Hip Extension (°)	10.2 (12.0)	9.5 (flex)–33.3
Swing: Peak Knee Flexion (°)	69.1 (13.0)	46.6–94.3
Stance: Peak Knee Flexion (°)	50.6 (16.2)	21.9–73.9
Swing: Peak Ankle PF (°)	12.2 (9.1)	4.6–29.4
Swing: Peak Ankle DF (°)	14.7 (5.6)	4.7–24.4
Toe-off: Peak Ankle PF (°)	11.0 (7.4)	0.5–25.6
Stance: Peak Ankle DF (°)	13.8 (6.1)	3.0–23.4

FTS: Floor to stand; STS: Sit to Stand; DF: Dorsiflexion; PF: Plantar Flexion; 3DMA: three-dimensional motion analysis.

## Data Availability

Data will be made available from the authors at reasonable request.
